# The Impact of the Coexpression of *MET* and *ESR* Genes on Prognosticators and Clinical Outcomes of Breast Cancer: An Analysis for the METABRIC Dataset

**DOI:** 10.1155/2024/2582341

**Published:** 2024-05-09

**Authors:** Nehad M. Ayoub, Ghaith M. Al-Taani, Amer E. Alkhalifa, Dalia R. Ibrahim, Aymen Shatnawi

**Affiliations:** ^1^Department of Clinical Pharmacy, Faculty of Pharmacy, Jordan University of Science and Technology, P.O. BOX: 3030, Irbid 22110, Jordan; ^2^Department of Clinical Pharmacy and Pharmacy Practice, Faculty of Pharmacy, Yarmouk University, Irbid, Jordan; ^3^Department of Drug Discovery and Biomedical Sciences, College of Pharmacy, Medical University of South Carolina, 70 President St., Charleston, SC 29425, USA

## Abstract

**Purpose:**

Breast cancer is a heterogeneous disease. Exploring new prognostic and therapeutic targets in patients with breast cancer is essential. This study investigated the expression of MET, ESR1, and ESR2 genes and their association with clinicopathologic characteristics and clinical outcomes in patients with breast cancer.

**Methods:**

The METABRIC dataset for breast cancer was obtained from the cBioPortal public domain. Gene expression data for *MET*, *ESR1*, and *ESR2*, as well as the putative copy number alterations (CNAs) for *MET* were retrieved.

**Results:**

The *MET* mRNA expression levels correlated inversely with the expression levels of *ESR1* and positively with the expression levels of *ESR2* (*r* = −0.379, *p* < 0.001 and *r* = 0.066, and *p*=0.004, respectively). The *ESR1* mRNA expression was significantly different among *MET* CNAs groups (*p* < 0.001). Patients with high *MET*/*ESR1* coexpression had favorable clinicopathologic tumor characteristics and prognosticators compared to low *MET/ESR1* coexpression in terms of greater age at diagnosis, reduced Nottingham Prognostic Index, lower tumor grade, hormone receptor positivity, HER2-negative status, and luminal subtype (*p* < 0.001). In contrast, patients with high *MET*/*ESR2* coexpression had unfavorable tumor features and advanced prognosticators compared to patients with low *MET*/*ESR2* coexpression (*p* < 0.001). No significant difference in overall survival was observed based on the *MET/ESR* coexpression status. However, when data were stratified based on the treatment type (chemotherapy and hormonal therapy), survival was significantly different based on the coexpression status of *MET/ESR*.

**Conclusions:**

Findings from our study add to the growing evidence on the potential crosstalk between MET and estrogen receptors in breast cancer. The expression of the MET/ESR genes could be a novel prognosticator and calls for future studies to evaluate the impact of combinational treatment approaches with MET inhibitors and endocrine drugs in breast cancer.

## 1. Introduction

Breast cancer is a heterogeneous disease that is classified based on gene expression profiles into the following five molecular subtypes: normal-like, luminal A, luminal B, human epidermal growth factor receptor 2 (HER2)-overexpressing, and basal-like tumors [1, 2]. The heterogeneity of breast cancer is both intertumoral and intratumoral. Intertumoral heterogeneity determines the differences encountered from one patient to another, while intratumoral one is explained by the diversity of tumor cell populations within the same primary tumor lesion [2]. The different molecular subtypes are associated with different clinical outcomes, treatment strategies, and prognostic values [2, 3]. Understanding the heterogeneity of breast cancer will improve the personalized care of patients and improve treatment outcomes.

Classic estrogen receptors (ERs) are members of the nuclear receptor superfamily of transcription regulators known to modulate gene expression in target tissues [4, 5]. Two types of ERs belong to the family of transcription factors, ER*α* and ER*β* [5]. The full-length human ER*α* is composed of 595 amino acids (67 kDa) and is encoded by the gene ESR1, located on chromosome 6 [4]. ER*α* is largely expressed in the mammary epithelium and plays a dominant role in mammary gland development as well as in breast cancer progression [5, 6]. Almost two thirds of breast cancer cases are ER*α*-positive [7]. The human ER*β* is encoded by the ESR2 gene located on chromosome 14 and comprises 530 amino acids (59 kDa) [4, 5]. ER*β* is abundant in normal breast epithelial cells and the rate of ER*β* positive expression in breast cancer has been reported to exceed 60% [8]. The ligand-binding domain and DNA-binding domain of the ER*β* protein are 60% and 96% homologous with those of ER*α* [8]. This finding suggests that both receptors may have similar but not identical functions [8]. The impact of ER*β* expression on mammary gland development and breast cancer is inconclusive [8]. The expression of ER*β* has been shown to suppress breast cancer cell proliferation and invasion [5, 6]. The antiproliferative effects of ER*β* are attributed, in part, to its ability to inhibit ER*α* selective target gene expression in breast tissue [5]. ER*β* isoforms that lack a known transcriptional activity can dimerize with ER*α* to suppress receptor signaling [4].

MET (c-MET) is a receptor tyrosine kinase (RTK) that belongs to the same family which includes Receptor d'Origine Nantais (RON) and ROS1 [9]. The MET proto-oncogene is located on chromosome 7 band 7q21–q31 [9]. The hepatocyte growth factor is the natural ligand of MET [10]. MET activation triggers an intricate genetic program known as “invasive growth,” leading to cell proliferation, invasion, angiogenesis, morphogenesis, and branching tubulogenesis [9, 10]. Deregulations of the MET signaling pathway are frequently encountered in several types of solid cancers. MET tyrosine kinase can be constitutively activated by mutation or amplification of the gene leading to overexpression and sustained receptor signaling in human cancers [9, 10]. The oncogenic activation of MET drives aggressive behavior including cancer cell proliferation, survival, scattering, epithelial-to-mesenchymal transition, invasion, and metastasis [9, 11]. In breast cancer, MET overexpression is detected in 20%–30% of all cases and 52% of triple-negative tumors [12]. Elevations of the *MET* copy number were reported in 8% of early breast cancer, mostly triple negative [13]. In addition, *MET* amplification and mutation were detected in 4.7% and 9% of advanced breast cancer patients, respectively [14]. There is no consensus on the prognostic impact of MET in breast cancer. While some studies indicated the association between higher MET expression and advanced tumor features, recurrence, and poor prognosis [15, 16], others revealed no association [13, 17].

Numerous studies have demonstrated the signaling crosstalk between ERs and RTK pathways. Such studies provided evidence that the activation of RTKs such as the epidermal growth factor receptor, HER2, and insulin-like growth factor receptor leads to the activation of ER in breast cancer independent of its ligand, thus promoting cancer cell survival and conferring resistance to antiestrogen therapy [18, 19]. Nevertheless, the crosstalk between ER and MET has not been well characterized with a limited number of studies exploring the association between ER and MET in human cancers. A meta-analysis of 6010 breast cancer cases indicated the association between MET overexpression and poor relapse-free survival in hormone receptor-positive disease [11]. Also, in patients with ER-positive/HER2-negative early breast cancer, high MET expression correlated with poor survival outcomes, suggesting its prognostic impact in patients with hormone-dependent tumors [20]. Previous data demonstrated that the overexpression of MET induced resistance to endocrine drug fulvestrant in breast cancer cell lines, an effect that was further associated with increased cancer cell migration and invasion [21]. Further evidence showed that pharmacologic inhibition of MET reversed resistance of the endocrine drugs in breast cancer cell lines [21, 22]. In addition, Vendrell et al. revealed that reduced expression of *ESR1* was associated with tamoxifen failure, disease relapse, and shorter overall survival (OS) in ER-positive breast tumor samples [23]. In line with this, the levels of *MET* mRNA were significantly higher in patients who failed tamoxifen treatment compared to those responding to tamoxifen [23]. Although the individual roles of ERs and MET in breast cancer have been illustrated, there remains limited knowledge regarding their coexpression and the resultant impact on the clinicopathologic features and prognosis of the disease. In light of this, we aimed to assess the expression pattern of ER genes, *ESR1* and *ESR2*, with *MET* and further explore their association with clinicopathologic features, prognostic factors, and clinical outcomes in breast cancer. Our approach is designed to further delineate tumor heterogeneity by exploring complex molecular interactions in breast cancer to expand our understanding of tumor behavior and introduce new therapeutic avenues that could further stratify patients into treatment groups based on their gene expression signatures.

## 2. Methods

### 2.1. The Molecular Taxonomy of the Breast Cancer International Consortium (METABRIC) Dataset

The METABRIC dataset provides clinical and genomic data on breast tumors from five different hospitals and/or research centers in the United Kingdom and Canada [24]. It allows the utilization of the molecular profiles of breast tumors to analyze the association with clinical outcomes and to better understand the clinical heterogeneity of the disease [24]. The METABRIC dataset was obtained for 2509 patients with primary breast cancer and downloaded from the cBio cancer genomics portal (cBioPortal). The cBioPortal is an open-access resource for interactive exploration of multidimensional cancer genomics datasets [25]. The METABRIC dataset provides demographic and clinical data such as the age of the patient as diagnosis, menopausal status, Nottingham Prognostic Index (NPI), OS, tumor histological subtype, the number of positive lymph nodes, tumor size, the TNM-stage, grade, receptor status, and molecular subtype. The treatment modality received by patients (type of breast surgery, hormonal therapy, chemotherapy, and/or radiotherapy) is also indicated in the dataset.

The METABRIC dataset includes microarray gene expression profile analysis and putative copy number alterations (CNAs) for several genes available in the dataset. Gene expression data for *MET*, *ESR1,* and *ESR2* along with *MET* CNAs were obtained from the dataset. mRNA gene expression log intensity values were available for 1904 out of the 2509 patients included and were used in this analysis. Values of CNAs were −2: homozygous deletion, −1: hemizygous deletion, 0: neutral (no change), 1: gain, and 2: high-level amplification.

### 2.2. Data Preprocessing

**Figure 1:**
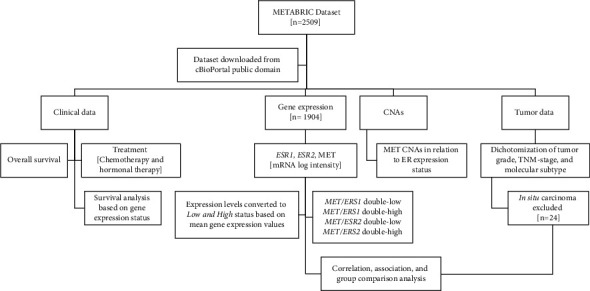
Study flowchart. CNAs: copy number alterations; ER: estrogen receptor.

Rigorous data preprocessing steps were taken before the statistical analysis. The downloaded demographic, clinical, and tumor data were carefully cleaned, coded, and uploaded to the statistical software. Afterwards, data cleansing was performed to rectify any inconsistencies, entry errors, or duplicate records. To be able to perform association analysis, the continuous gene expression data were converted to categorical data. The expression of each gene was divided into low and high expression categories based on the mean expression value, in which patients with mRNA expression levels equal to or less than the mean value were indicated to have a low expression status, while those with expression levels greater than the mean value were set to have a high expression status. In addition, the dichotomization of some categorical variables was considered for the association analysis and was performed in advance of conducting statistical analysis to avoid a small sample size [26]. Therefore, the tumor grade was categorized as grade I/II and grade III. The TNM stage was dichotomized as early (stage I/II) and advanced (stage III/IV), excluding patients with noninvasive tumors (*in situ* carcinoma). The molecular subtype was grouped as a luminal (luminal A and luminal B) and non-luminal (normal-like, HER2-positive, basal-like, and claudin-low) disease. The categories of these tumor variables were selected using cut points previously reported [26]. For conducting the survival analysis, patients with missing survival status, survival time, or the expression of *ESR1*, *ESR2*, and/or *MET* were excluded from the analysis. A flowchart for the study is shown in Figure 1.

### 2.3. Statistical Analysis

Data analysis was performed using the SPSS statistical package, version 23.0 (IBM Corp, Armonk, NY). Continuous variables are presented as the mean ± standard deviation and categorical variables are presented as frequencies and percentages. To assess the correlations between the continuous variables, Pearson's correlation test was applied. An independent sample *t*-test was used to compare the mean of two groups. One-way analysis of variance (ANOVA) was used for comparisons between multiple independent groups followed by Tukey's post hoc analysis. To assess associations between categorical variables, the chi-square test of independence was applied. Kaplan–Meier survival curves were generated for patients according to the gene expression status using GraphPad Prism, version 8.0.1, software (GraphPad Software, San Diego, CA). Cox proportional hazards models were fitted with OS as the outcome. All *p* values were two sided, and differences were considered statistically significant at *p* < 0.05.

## 3. Results

### 3.1. Demographic and Clinicopathologic Characteristics of Patients with Breast Cancer in the MEATBRIC Dataset

A description of the demographic and clinicopathologic characteristics of the METABRIC dataset was previously described [26].

### 3.2. The Expression of *MET* Correlates with *ESR1* and *ESR2* in Patients with Breast Cancer

**Figure 2:**
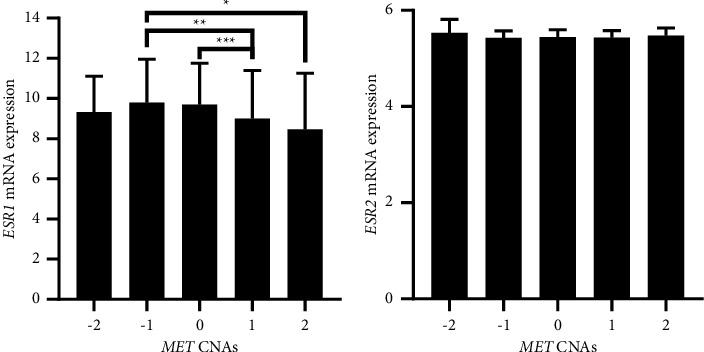
The level of *ESR* mRNA expression based on *MET* CNAs in patients with breast cancer. The mRNA expression of (a) *ESR1* and (b) *ESR2* according to *MET* CNAs. Values of CNAs are −2: homozygous deletion, −1: hemizygous deletion, 0: neutral (no change), 1: gain, and 2: high-level amplification. One-way ANOVA, ^*∗*^*p* < 0.05, ^*∗∗*^*p* < 0.01, and ^*∗∗∗*^*p* < 0.001. ns: no statistically significant difference. Bars represent mean mRNA gene expression log intensity ± standard deviation. CNAs: copy number alterations.

**Figure 3:**
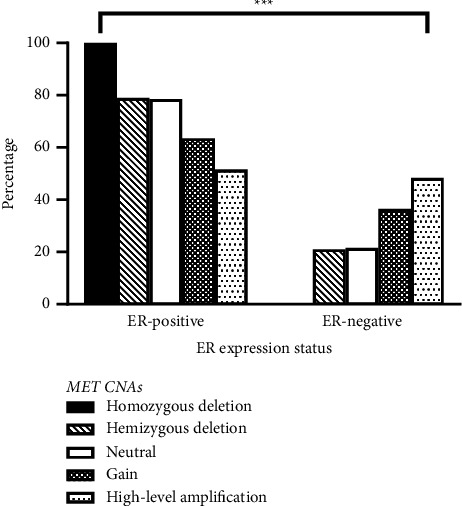
*MET* CNAs based on the ER status in patients with breast cancer. Bars represent the percentage of patients with breast cancer within each group of ER expression status and the CNAs. Chi-square test and ^*∗∗∗*^*p* < 0.001. CNAs: copy number alterations; ER: estrogen receptor.

Bivariate correlation analysis revealed that *MET* mRNA expression levels correlated inversely with *ESR1* mRNA levels in patients (*r* = − 0.379, *p* < 0.001). Alternatively, *MET* expression correlated positively with *ESR2* (*r* = 0.066, *p*=0.004). However, the correlation between *MET* and *ESR1* was stronger than *ESR2*. In this study, 2138 patients had *MET* CNAs data, of whom 3 (0.1%) had homozygous deletion, 206 (9.6%) had hemizygous deletion, 1661 (77.7%) had no change, 237 (11.1%) had gain, and 31 (1.4%) had high-level amplification. One-way ANOVA revealed that *ESR1* mRNA expression was significantly different among *MET* CNAs groups (*F* = 7.16, *p* < 0.001, Figure 2(a)). The *ESR1* expression was significantly lower in patients with *MET* high-level amplification than with hemizygous deletion (*p*=0.044, Figure 2(a)). In addition, patients with *MET* gain had significantly lower *ESR1* mRNA expression levels compared to patients with no change (*p* < 0.001) and those with hemizygous deletion (*p*=0.002). Alternatively, *ESR2* mRNA expression was not significantly different among *MET* CNAs (*F* = 1.07, *p*=0.371, Figure 2(b)). Besides, *MET* CNAs are significantly associated with ER expression status (*p* < 0.001, Figure 3). In patients with the ER-positive status, high-level amplification was the least observed *MET* CNA (51.6%). Alternatively, high-level amplification was the most common *MET* CNA in patients with the ER-negative status (48.4%). None of the patients with the ER-negative status had *MET* homozygous deletion (Figure 3).

### 3.3. The Impact of *MET/ESR* Coexpression on Demographic and Clinicopathologic Characteristics in Patients with Breast Cancer

Table 1 describes mRNA expression log intensity for the three genes in breast cancer patients. In this cohort, 1087 patients (57.1%) had low *MET* expression and 713 (37.4%) and 986 (51.8%) had low *ESR1* and *ESR2* mRNA expression, respectively (Table 1). Patients were stratified into 8 groups based on the expression status of MET and ESR genes. Most patients had a *MET*^Low^/*ESR*1^High^ coexpression status (41.3%) while *MET*^Low^/*ESR*1^Low^ was least prevalent (15.7%) (Table 1). The double-low and double-high expression of both MET and ESR genes were considered for further analysis in this study.

**Figure 4:**
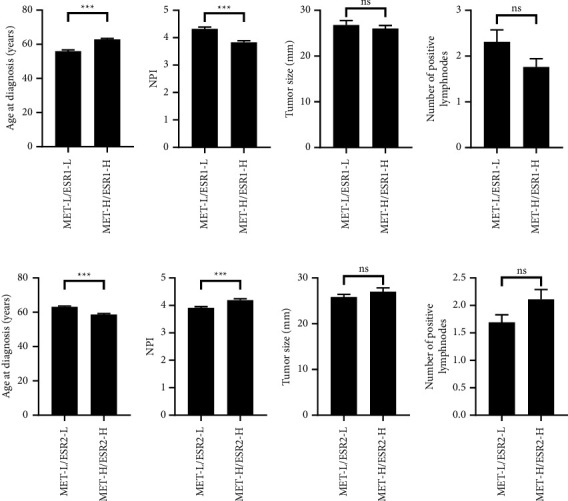
The effect of *MET/ESR* coexpression on demographic and tumor characteristics in patients with breast cancer. Comparison of *MET/ESR1* coexpression according to (a) age at diagnosis, (b) NPI, (c) tumor size, and (d) the number of positive lymph nodes and a comparison of *MET/ESR2* coexpression according to (e) age at diagnosis, (f) NPI, (g) tumor size, and (h) number of positive lymph nodes. Independent-sample *t*-test and ^*∗∗∗*^*p* < 0.001. ns: no statistically significant difference. H: high; L: low; NPI: Nottingham Prognostic Index.

Next, we evaluated the impact of the MET/ESR gene coexpression on demographic and clinicopathologic features. Patients with *MET*^High^/*ESR*1^High^ status had a significantly higher mean age at diagnosis and a lower NPI than patients within the *MET*^Low^/*ESR*1^Low^ group (Figures 4(a) and 4(b), *p* < 0.001). This pattern was reversed in the case of the *MET/ESR2* coexpression in which patients with the *MET*^High^/*ESR*2^High^ status had significantly lower age and greater NPI compared to patients with *MET*^Low^/*ESR*2^Low^ coexpression (Figures 4(e) and 4(f), *p* < 0.001). The tumor size and the number of positive lymph nodes were not different according to the coexpression status of *MET/ESR* (Figure 4).

The association between *MET/ESR* coexpression and other clinicopathologic features of breast cancer patients is shown in Table 2. Compared to the *MET*^Low^/*ESR*1^Low^ status, the *MET*^High^/*ESR*1^High^ coexpression was significantly associated with grade I/II tumors, hormone receptor positivity, HER2-negative status, and luminal disease (*p* < 0.001). On the contrary, *MET*^High^/*ESR*2^High^ coexpression was significantly associated with advanced stage, high-grade, hormone receptor-negativity, and non-luminal subtype compared to patients with *MET*^Low^/*ESR*2^Low^ coexpression (*p* < 0.001, Table 2).

### 3.4. The Impact of *MET/ESR* Coexpression on Overall Survival and Treatment Outcomes in Patients with Breast Cancer

**Figure 5:**
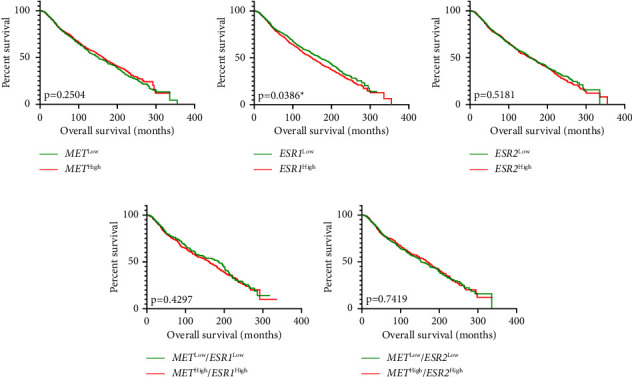
Overall survival rate of patients with breast cancer based on gene expression. Kaplan–Meier survival analyses based on (a) *MET*, (b) *ESR1*, (c) *ESR2*, (d) *MET/ESR1*, and (e) *MET/ESR2* coexpression. Log-rank (Mantel–Cox) test. ^*∗*^Indicates statistical significance at *p* < 0.05.

Survival analysis showed that patients with low *ESR1* expression had significantly longer OS compared to patients with high *ESR1* expression (*p*=0.0386, 95% CI = 0.7744–0.9913, Figure 5(b)). Alternatively, the expression status of *MET* and *ESR2* did not affect the survival of patients (Figure 5(a) and 5(c)). Upon comparing survival curves based on the coexpression status, no significant difference in OS was observed for *MET/ESR1* and *MET/ESR2* coexpression groups (Figures 5(d) and 5(e)).

**Figure 6:**
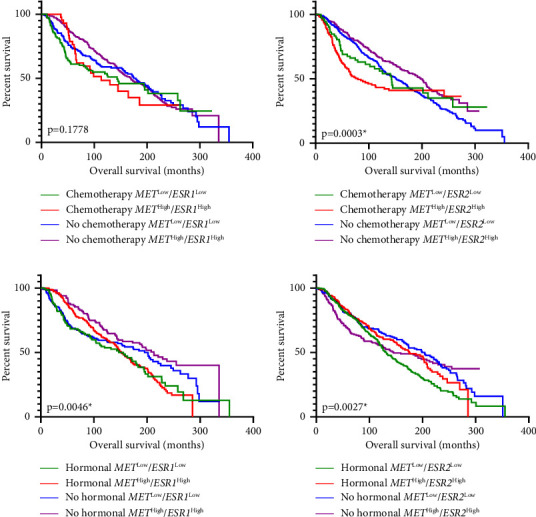
Overall survival rate of patients with breast cancer based on *MET*/*ESR* gene coexpression and the type of treatment. Kaplan–Meier survival analyses based on chemotherapy treatment in (a) *MET*/*ESR1*, (b) *MET*/*ESR2*, and hormonal treatment in (c) *MET/ESR1*, and (d) *MET/ESR2* coexpression. Log-rank (Mantel–Cox) test. ^*∗*^Indicates statistical significance at *p* < 0.05.

The impact of the coexpression of *MET/ESR* on OS was further analyzed according to the type of treatment as shown in Figure 6. Compared with high coexpression, low *MET/ESR1* coexpression was associated with longer median survival time whether chemotherapy was received or not; however, these differences did not reach statistical significance (*p*=0.1778, Figure 6(a)). In patients who received chemotherapy, those with low *MET/ESR2* coexpression had higher median survival compared to those with high coexpression (median survival 142.6 vs. 79.4 months, respectively, Figure 6(b)). Alternatively, in patients who did not receive chemotherapy, median survival was greater for those with high *MET/ESR2* coexpression compared with low coexpression (median survival 197.7 vs. 151.2 months, respectively, *p*=0.0003, Figure 6(b)). Patients with high *MET/ESR1* coexpression had a longer median survival time compared to those with a low coexpression whether hormonal treatment was administered or not (*p*=0.0046, Figure 6(c)). Patients with high *MET/ESR2* coexpression who administered hormonal drugs had a longer survival time compared to those with low *MET/ESR2* coexpression (median survival 175.1 vs. 132.03 months, respectively, *p*=0.0027, Figure 6(d)). However, in the absence of hormonal treatment, patients with low *MET/ESR2* coexpression had longer survival compared to those with high *MET/ESR2* coexpression (median survival 204.2 vs. 145.4 months, respectively, Figure 6(d)).

## 4. Discussion

Despite the expanding number of new anticancer agents, treatment failure, relapse, and progression remain major challenges in the management of breast cancer [3, 27]. Therefore, there is an urgent need to identify new prognostic factors and therapeutic targets to improve treatment outcomes in breast cancer [27, 28]. ER*α* is a nuclear receptor expressed in almost 70% of breast cancers and a key driver of carcinoma initiation and proliferation in hormone-dependent tumors [29]. It promotes the expression of oncogenic proteins that enhance cancer cell growth, survival, and progression such as cyclin D1, c-Myc, and insulin-like growth factor 1, while inhibiting cell cycle arrest proteins such as p21 [7, 29]. In addition, ER*α* modulates the expression of genes that regulate breast cancer cell migration and metastasis [6]. On the other hand, the role of ER*β* in breast cancer is less established. ER*β* has an antiproliferative activity when introduced into ER*α*-positive breast cancer cells [30]. The expression of ER*β* was associated with reduced proliferation and invasion of breast tumors [6]. The molecular pathways associated with the antiproliferative effects of ER*β* are less clear; however, ER*β* has been shown to reduce the expression of c-Myc while inducing the expression of the cyclin-dependent kinase inhibitor, p27Kip1, in breast cancer cells [30]. MET is an RTK commonly expressed in epithelial cells and is highly implicated in tumorigenesis. MET is expressed in different molecular subtypes of breast cancer and is associated with aggressive phenotypes. In this study, we assessed the coexpression of MET and ESR genes and their impact on tumor features and treatment outcomes in breast cancer.

In this study, the mean mRNA expression levels for *ESR1* were higher than *MET* and *ESR2*. Besides, the mRNA levels of *MET* correlated inversely with *ESR1* and positively with *ESR2* mRNA levels. *MET* CNAs were also associated with the ER-expression status in patients. A reduced expression of *ESR1* in patients with *MET* gain and high-level amplification compared to those with no change or hemizygous deletion was observed in this analysis. However, *MET* CNAs did not affect the mRNA levels of *ESR2*. In this context, few studies have evaluated the expression of MET and ER in cancer. In agreement with our findings, Ren et al. revealed that MET and ER*β* were overexpressed in basal-like breast cancer and MET overexpression was associated with ER*β* positivity [10]. In addition, Tao et al. indicated that ER*β* positively regulated *MET* expression in bladder cancer [31]. Findings from cell culture and animal models showed that ER*β* signaling is induced by infiltrating T cells and consequently increased MET expression directly by binding to MET gene promoter or through modulation of interleukin-1 expression in bladder cancer, leading to invasion and metastasis. Such findings could explain the positive correlation between the expression of the ESR2 and MET genes observed in breast cancer in our study, particularly in the more immunogenic non-liminal subtypes. Mast cells have been shown to induce the expression and activation of *ESR1* and its target genes promoting luminal phenotype while concomitantly suppressing the activation of MET in breast cancer using in vivo and in silico models [32]. In endometrial carcinomas, the expression of ER and MET correlated inversely and the coexpression of both receptors predicted response to hormonal therapy [18]. Together, the expression of the MET gene could be regulated by ERs in cancer cells. The regulation is determined by the type of ER, in which ER*α* downregulates while ER*β* upregulates the expression of MET. The regulation of MET expression by ERs could be indirect as well, involving other pathways. Nevertheless, a crosstalk between RTKs and ERs has been previously indicated and MET could be a target for ERs. The expression patterns shown in our analysis allow a better understanding of the impact of MET on the development and progression of breast cancer.

Our findings revealed that high *MET/ESR1* coexpression was associated with favorable clinicopathologic tumor features such as greater age at diagnosis, lower NPI scores, low-grade carcinoma, hormone receptor positivity, HER2-negative status, and luminal subtype. Interestingly, the opposite pattern was observed for the *MET/ESR2* coexpression in which high coexpression was associated with younger age at diagnosis, increased NPI, advanced stage and grade of carcinoma, hormone receptor-negative status, and non-luminal tumors. These findings indicate different roles for the ESR genes and their influence on tumor characteristics and prognosticators. Furthermore, the impact of the MET gene on disease characteristics was dependent on its partner ESR gene in our study, and it revealed a striking difference when *MET* expression was associated with *ESR1* or *ESR2*. Together, these findings provide a rationale for risk stratification and treatment of breast cancer patients based on gene expression of *MET* and *ESR*. The increased expression of MET/ESR2 genes in younger patients with breast cancer may explain the aggressive phenotype and the advanced tumor characteristics that collectively impose a worse prognosis in this group of patients. Previous studies have investigated the coexpression of MET and other target receptors in breast cancer and their impact on disease presentation and prognosis. Baccelli et al. showed that the coexpression of MET and CD47, a ligand involved in cancer cell evasion from macrophage scavenging was strongly associated with lymph node metastasis [33]. The coexpression of MET and plexin-B1, the receptor of Sema4D, was associated with advanced-stage breast and ovarian carcinoma as indicated in MET/plexin-B1 double-positive tumors. Furthermore, tumors coexpressing MET/plexin-B1 had a higher grade and incidence of lymph node metastases [34]. These findings are particularly important in revising the classical prognosticators in breast cancer which relied on the expression status of ER*α*, progesterone receptor, and HER2 as the main receptors for classifying patients into distinct molecular subtypes and predicting a response to therapy. The heterogeneity of breast cancer calls for a deeper analysis of other tumor biomarkers that could further influence the prognosis of patients. Considering the complexity of the disease, expanding the number of biomarkers, and using a combination of them could better serve this goal.

The expression of ER*α* has both predictive and prognostic values; however, it mainly indicates the eligibility of patients for endocrine therapy [35, 36]. The prognostic impact of ER*β* protein in patients with breast cancer is less clear [8]. While some studies revealed an association between the expression levels of ER*β* and OS [37, 38], other studies revealed worse outcomes or a lack of association [8, 39]. The MET expression correlated with poor prognosis in several studies in patients with breast cancer. High MET levels were associated with an increased risk of disease recurrence and reduced OS [15, 40]. Alternatively, other studies showed MET expression to be associated with a favorable prognosis and increased survival in breast cancer patients [41]. In our study, *ERS1* was associated with OS in patients, and those with low gene expression had significantly prolonged survival compared to high expression cases. Alternatively, the expression of *MET* and *ESR2* was not associated with OS in our study. Similarly, Ren et al. showed that the overexpression of MET and ER*β* was not associated with recurrence or mortality in patients with basal-like breast cancer [10]. In addition, no difference in OS was observed when comparing the double-low to the double-high expression of both *MET/ESR1* and *MET/ESR2*. Nevertheless, our results revealed a prolonged OS for patients treated with chemotherapy and coexpressing *MET/ESR2* low status compared to those who did not receive chemotherapeutic drugs. Alternatively, survival was longer for patients who received hormonal treatment and had high *MET/ESR2* coexpression compared to those with low coexpression. Thus, the coexpression status of *MET* and *ESR2* could predict a response to treatment in patients with breast cancer. In a study by Lee et al., the 10-year disease-free survival in patients with MET-negative/RON-negative breast tumors was 79.3% compared to 11.8% in patients with MET-positive/RON-positive tumors [42]. A 10.3-year difference in mean OS was shown between MET/CD47 double-positive and double-negative breast cancer patients who had hormone receptor-positive tumors [33]. In a recent study by Motomura et al., high coexpression of MET with aldehyde dehydrogenase and protein kinase C was associated with an advanced stage and poor prognosis in patients with breast cancer compared with low coexpression [43].

Endocrine therapies such as selective estrogen receptor modulators and aromatase inhibitors are the cornerstone treatment for breast cancer patients with hormone-dependent tumors [29]. Despite the well-known activity of endocrine drugs, the rates of de novo or acquired resistance are rising, thus limiting the clinical effectiveness of such therapy [28, 44]. The majority of ER*α*-positive tumors will develop acquired resistance to hormonal drugs without any alteration in their ER profile [45]. A proposed mechanism of resistance to endocrine therapy is signaling crosstalk between ER and oncogenic RTKs [7, 29]. MET has been shown to mediate endocrine drug resistance in breast cancer [21, 22]. Jaeger et al. showed a synergistic anticancer effect for the combination of raloxifene with the MET inhibitor cabozantinib in hormone receptor-positive breast cancer cells [3]. In addition, recent studies from our lab demonstrated remarkable synergistic anticancer activity for the combination of crizotinib, a MET inhibitor, with the endocrine drugs tamoxifen and fulvestrant in different breast cancer cell lines in vitro [46, 47]. Taken together, MET could be an appealing target for the treatment of hormone-dependent breast cancer. Findings from this study highlight the importance of molecular ER subtyping at the diagnosis of breast cancer along with the MET expression status. The expression profile could point out patients who are at higher risk of poor outcomes based on the panel of prognostic factors analyzed in this study. The availability of clinically approved MET inhibitors expands the treatment options to consider in patients with hormone-dependent tumors who lack response or develop resistance to their endocrine therapies. A high expression of MET and ER*β* could be a novel biomarker to investigate among patients with limited response to drugs targeting ER*α*. In this regard, the clinical usefulness of MET inhibitors in patients harboring breast tumors with high ER*β* expression is worth investigating. Nevertheless, translating our findings into clinical settings could be faced with several challenges, most importantly the development of standardized gene expression profiling tools and protocols for the coexpression of *MET* and *ESRs*. In addition, the low possibilities of gene testing signatures of ERs in the early diagnosis phase and the lack of standardized immunohistochemical staining assays for MET could add another layer of complexity [48]. Besides, the feasibility, cost, and turn-around time of such investigations must be addressed to ensure that patients can benefit from these insights promptly.

Despite the insightful findings, our study has some limitations. Our findings are based on the mRNA expression levels of the ESR and MET genes. While the mRNA levels are informative, they do not necessarily correlate with protein expression levels and function, which are ultimately responsible for the phenotypic outcomes in cancer. Hence, our results should be interpreted with caution, acknowledging that mRNA expression may not fully capture the complex regulation and activity of ERs and MET in breast cancer. In addition, the observational design of our study limits the ability to infer causality between the gene expression patterns and the clinical outcomes. Future research should address these limitations. Prospective, multicenter studies are essential to confirm our findings and ensure their applicability across diverse patient populations. Moreover, integrating proteomic analyses with genomic data could provide a more comprehensive understanding of the role of ERs and MET protein levels and their functional interactions in breast cancer. Longitudinal studies that monitor changes in *MET* and *ESR* expressions during treatment would offer valuable insights into their roles as potential biomarkers for treatment response and disease progression. Alongside, preclinical studies can help understand the role of MET inhibitors in models of breast cancer and analyze the role of MET in responding to endocrine treatment to pave the way for clinical trials to explore the usefulness of combinational treatment approaches of MET inhibitors and hormonal therapies.

## 5. Conclusions

**Figure 7:**
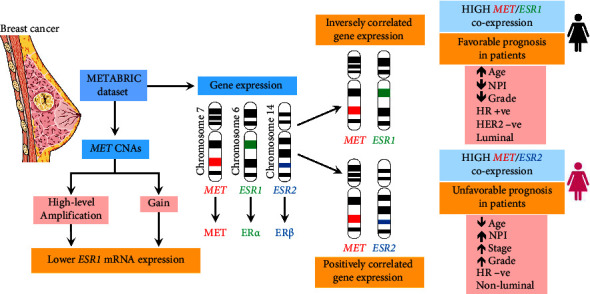
A summary of the main findings of the current study. The figure was partially created using free medical images available from servier medical art (smart.servier.com). CNAs: copy number alterations; ER: estrogen receptor; HER2: human epidermal growth factor receptor 2; HR: hormone receptor; NPI: Nottingham Prognostic Index.

Due to the complexity and heterogeneity of breast cancer, exploring new prognostic factors and therapeutic targets is of paramount importance. To our knowledge, this is the first study to analyze the association of *MET* and *ESR* expressions in breast cancer. Our findings revealed distinct coexpression patterns of *MET/ESR1* and *MET/ESR2*, each correlating with specific clinicopathologic features and clinical outcomes, thereby enriching the prognostic landscape of breast cancer. Importantly, we demonstrate the potential of *MET/ESR* coexpression as a robust prognostic tool, especially in predicting survival outcomes for patients undergoing chemotherapy or hormonal therapy. Our study calls for a comprehensive re-evaluation of the impact of ER*β* in breast cancer, which could influence the treatment approaches and prognostic assessment of the disease. Future research will be needed to deepen our understanding of the molecular interplay between ER and MET to integrate these markers into clinical decision-making processes towards personalizing breast cancer care.  Figure 7 summarizes the main findings from this study.

## Figures and Tables

**Table 1:** *MET*, *ESR1*, and *ESR2* mRNA expressions in patients with breast cancer.

Characteristics	Mean ± SD (range)

*MET* mRNA expression log intensity	5.61 ± 0.29 (4.96–7.86)
*ESR1* mRNA expression log intensity	9.61 ± 2.13 (5.22–13.27)
*ESR2* mRNA expression log intensity	5.44 ± 0.15 (4.90–6.28)

Characteristics	*n* (%)

*MET* mRNA^†^	
Low	1087 (57.1)
High	817 (42.9)
*ESR1* mRNA^†^	
Low	713 (37.4)
High	1191 (62.6)
*ESR2* mRNA^†^	
Low	986 (51.8)
High	918 (48.2)
*MET/ESR* coexpression status	
*MET*^Low^/*ESR*1^Low^	299 (15.7)
*MET*^Low^/*ESR*1^High^	786 (41.3)
*MET*^High^/*ESR*1^Low^	416 (21.8)
*MET*^High^/*ESR*1^High^	403 (21.2)
*MET*^Low^/*ESR*2^Low^	591 (31.0)
*MET*^Low^/*ESR*2^High^	498 (26.2)
*MET*^High^/*ESR*2^Low^	363 (19.1)
*MET*^High^/*ESR*2^High^	452 (23.7)

^†^Patients with mRNA gene expression equal to or below the mean value were indicated to have “low” expression status, while those with mRNA log intensity greater than the mean value were set to have “high” expression status. *n*(%); frequency and valid percentage.

**Table 2:** Association of the *MET*/*ESR* gene expression status with clinicopathologic characteristics in patients with breast cancer.

Parameter	Expression status		*p* value	Expression status		*p* value
	*MET* ^Low^/*ESR*1^Low^ (*n* = 299)	*MET* ^High^/*ESR*1^High^ (*n* = 403)		*MET* ^Low^/*ESR*2^Low^ (*n* = 591)	*MET* ^High^/*ESR*2^High^ (*n* = 452)	
Stage			0.746			0.001^*∗*^
Early	191 (40.6)	280 (59.4)		411 (58.1)	297 (41.9)	
Advanced	14 (37.8)	23 (62.2)		27 (37.5)	45 (62.5)	
Grade			<0.001^*∗*^			<0.001^*∗*^
I/II	92 (27.2)	246 (72.8)		311 (63.7)	177 (36.3)	
III	198 (58.1)	143 (41.9)		255 (49.5)	260 (50.5)	
ER			<0.001^*∗*^			<0.001^*∗*^
Positive	150 (28.0)	385 (72.0)		517 (63.7)	295 (36.3)	
Negative	146 (92.4)	12 (7.6)		65 (30.1)	151 (69.9)	
PR			<0.001^*∗*^			<0.001^*∗*^
Positive	86 (22.8)	291 (77.2)		372 (65.0)	200 (35.0)	
Negative	213 (65.5)	112 (34.5)		219 (46.5)	252 (53.5)	
HER2			<0.001^*∗*^			0.064
Positive	77 (76.2)	24 (23.8)		58 (48.7)	61 (51.3)	
Negative	222 (36.9)	379 (63.1)		533 (57.7)	391 (42.3)	
Molecular subtype			<0.001^*∗*^			<0.001^*∗*^
Luminal	70 (17.8)	323 (82.2)		458 (70.4)	193 (29.6)	
Non-luminal	229 (74.8)	77 (25.2)		132 (33.9)	257 (66.1)	

Data are presented as *n*(%). Chi-square test. ^*∗*^Indicates statistical significance at *p* < 0.05. ER: estrogen receptor; HER2: human epidermal growth factor receptor 2; PR: progesterone receptor.

## Data Availability

The MATABRIC dataset analyzed in this study is freely accessible in the cBioPortal public domain (available at: https://www.cbioportal.org/).

## References

[1] Yersal O., Barutca S. (2014). Biological subtypes of breast cancer: prognostic and therapeutic implications. *World Journal of Clinical Oncology*.

[2] Luond F., Tiede S., Christofori G. (2021). Breast cancer as an example of tumour heterogeneity and tumour cell plasticity during malignant progression. *British Journal of Cancer*.

[3] Jaeger S., Igea A., Arroyo R. (2017). Quantification of pathway cross-talk reveals novel synergistic drug combinations for breast cancer. *Cancer Research*.

[4] Fuentes N., Silveyra P. (2019). Estrogen receptor signaling mechanisms. *Advances in Protein Chemistry and Structural Biology*.

[5] Ayoub N. M., Victor R., Preedy R. R. W. (2021). Olive oil oleocanthal and estrogen receptor expression. *Olives and Olive Oil in Health and Disease Prevention*.

[6] Saha Roy S., Vadlamudi R. K. (2012). Role of estrogen receptor signaling in breast cancer metastasis. *International Journal of Breast Cancer*.

[7] Xue M., Zhang K., Mu K. (2019). Regulation of estrogen signaling and breast cancer proliferation by an ubiquitin ligase TRIM56. *Oncogenesis*.

[8] Zhou Y., Liu X. (2020). The role of estrogen receptor beta in breast cancer. *Biomarker research*.

[9] Ayoub N. M., Ibrahim D. R., Alkhalifa A. E. (2021). Overcoming resistance to targeted therapy using MET inhibitors in solid cancers: evidence from preclinical and clinical studies. *Medical Oncology*.

[10] Ren X., Yuan L., Shen S., Wu H., Lu J., Liang Z. (2016). c-Met and ER*β* expression differences in basal-like and non-basal-like triple-negative breast cancer. *Tumor Biology*.

[11] Yan S., Jiao X., Zou H., Li K. (2015). Prognostic significance of c-Met in breast cancer: a meta-analysis of 6010 cases. *Diagnostic Pathology*.

[12] Sapana Sameer Chaudhary S. C., Rawat S., Gouri A., Bilgrami A. L., Ghulam Md A., Gupta S. P. (2020). c-Met as a potential therapeutic target in triple negative breast cancer. *Cancer-Leading Proteases*.

[13] Zagouri F., Brandstetter A., Moussiolis D. (2014). Low protein expression of MET in ER-positive and HER2-positive breast cancer. *Anticancer Research*.

[14] de Melo Gagliato D., Jardim D. L., Falchook G. (2014). Analysis of MET genetic aberrations in patients with breast cancer at MD Anderson Phase I unit. *Clinical Breast Cancer*.

[15] Zagouri F., Bago-Horvath Z., Rossler F. (2013). High MET expression is an adverse prognostic factor in patients with triple-negative breast cancer. *British Journal of Cancer*.

[16] Zhao X., Qu J., Hui Y. (2017). Clinicopathological and prognostic significance of c-Met overexpression in breast cancer. *Oncotarget*.

[17] Gisterek I., Lata E., Halon A. (2011). Prognostic role of c-met expression in breast cancer patients. *Reports of Practical Oncology and Radiotherapy*.

[18] Pegram M., Jackisch C., Johnston S. R. D. (2023). Estrogen/HER2 receptor crosstalk in breast cancer: combination therapies to improve outcomes for patients with hormone receptor-positive/HER2-positive breast cancer. *NPJ Breast Cancer*.

[19] Britton D. J., Hutcheson I. R., Knowlden J. M. (2006). Bidirectional cross talk between ER*α* and EGFR signalling pathways regulates tamoxifen-resistant growth. *Breast Cancer Research and Treatment*.

[20] Iovino F., Diana A., Carlino F. (2022). Expression of c-MET in estrogen receptor positive and HER2 negative resected breast cancer correlated with a poor prognosis. *Journal of Clinical Medicine*.

[21] Hiscox S., Jordan N. J., Jiang W. (2006). Chronic exposure to fulvestrant promotes overexpression of the c-Met receptor in breast cancer cells: implications for tumour-stroma interactions. *Endocrine-Related Cancer*.

[22] Basak P., Chatterjee S., Bhat V. (2018). Long non-coding RNA H19 acts as an estrogen receptor modulator that is required for endocrine therapy resistance in ER+ breast cancer cells. *Cellular Physiology and Biochemistry*.

[23] Vendrell J. A., Robertson K. E., Ravel P. (2008). A candidate molecular signature associated with tamoxifen failure in primary breast cancer. *Breast Cancer Research*.

[24] Papatheodorou I., Crichton C., Morris L. (2009). A metadata approach for clinical data management in translational genomics studies in breast cancer. *BMC Medical Genomics*.

[25] Cerami E., Gao J., Dogrusoz U. (2012). The cBio cancer genomics portal: an open platform for exploring multidimensional cancer genomics data. *Cancer Discovery*.

[26] Shatnawi A., Ayoub N. M., Alkhalifa A. E. (2021). ING4 expression landscape and association with clinicopathologic characteristics in breast cancer. *Clinical Breast Cancer*.

[27] Ju J., Zhu A. J., Yuan P. (2018). Progress in targeted therapy for breast cancer. *Chronic Diseases and Translational Medicine*.

[28] Masoud V., Pages G. (2017). Targeted therapies in breast cancer: new challenges to fight against resistance. *World Journal of Clinical Oncology*.

[29] Zabransky D. J., Park B. H. (2014). Estrogen receptor and receptor tyrosine kinase signaling: use of combinatorial hormone and epidermal growth factor receptor/human epidermal growth factor receptor 2-targeted therapies for breast cancer. *Journal of Clinical Oncology*.

[30] Haldosen L. A., Zhao C., Dahlman-Wright K. (2014). Estrogen receptor beta in breast cancer. *Molecular and Cellular Endocrinology*.

[31] Tao L., Qiu J., Slavin S. (2018). Recruited T cells promote the bladder cancer metastasis via up-regulation of the estrogen receptor *β*/IL-1/c-MET signals. *Cancer Letters*.

[32] Majorini M. T., Cancila V., Rigoni A. (2020). Infiltrating mast cell-mediated stimulation of estrogen receptor activity in breast cancer cells promotes the luminal phenotype. *Cancer Research*.

[33] Baccelli I., Stenzinger A., Vogel V. (2014). Co-expression of MET and CD47 is a novel prognosticator for survival of luminal-type breast cancer patients. *Oncotarget*.

[34] Valente G., Nicotra G., Arrondini M. (2009). Co-expression of plexin-B1 and Met in human breast and ovary tumours enhances the risk of progression. *Analytical Cellular Pathology*.

[35] Cianfrocca M., Goldstein L. J. (2004). Prognostic and predictive factors in early-stage breast cancer. *The Oncologist*.

[36] Allred D. C. (2010). Issues and updates: evaluating estrogen receptor-*α*, progesterone receptor, and HER2 in breast cancer. *Modern Pathology*.

[37] Sugiura H., Toyama T., Hara Y. (2007). Expression of estrogen receptor *β* wild-type and its variant ER*β*cx/*β*2 is correlated with better prognosis in breast cancer. *Japanese Journal of Clinical Oncology*.

[38] Nakopoulou L., Lazaris A. C., Panayotopoulou E. G. (2004). The favourable prognostic value of oestrogen receptor beta immunohistochemical expression in breast cancer. *Journal of Clinical Pathology*.

[39] Guo L., Zhang Y., Zhang W., Yilamu D. (2014). Correlation between estrogen receptor *β* expression and the curative effect of endocrine therapy in breast cancer patients. *Experimental and Therapeutic Medicine*.

[40] Raghav K. P., Wang W., Liu S. (2012). cMET and phospho-cMET protein levels in breast cancers and survival outcomes. *Clinical Cancer Research*.

[41] Koh Y. W., Lee H. J., Ahn J. H., Lee J. W., Gong G. (2014). MET expression is associated with disease-specific survival in breast cancer patients in the neoadjuvant setting. *Pathology, Research & Practice*.

[42] Lee W. Y., Chen H. H., Chow N. H., Su W. C., Lin P. W., Guo H. R. (2005). Prognostic significance of co-expression of RON and MET receptors in node-negative breast cancer patients. *Clinical Cancer Research*.

[43] Motomura H., Nozaki Y., Onaga C. (2020). High expression of *c-met*, *PKCλ* and *ALDH1A3* predicts a poor prognosis in late-stage breast cancer. *Anticancer Research*.

[44] Baliu-Pique M., Pandiella A., Ocana A. (2020). Breast cancer heterogeneity and response to novel therapeutics. *Cancers*.

[45] Skandalis S. S., Afratis N., Smirlaki G. (2014). Cross-talk between estradiol receptor and EGFR/IGF-IR signaling pathways in estrogen-responsive breast cancers: focus on the role and impact of proteoglycans. *Matrix Biology*.

[46] Ayoub N. M., Ibrahim D. R., Alkhalifa A. E., Al-Husein B. A. (2021). Crizotinib induced antitumor activity and synergized with chemotherapy and hormonal drugs in breast cancer cells via downregulating MET and estrogen receptor levels. *Investigational New Drugs*.

[47] Ayoub N. M., Alkhalifa A. E., Ibrahim D. R., Alhusban A. (2021). Combined crizotinib and endocrine drugs inhibit proliferation, migration, and colony formation of breast cancer cells via downregulation of MET and estrogen receptor. *Medical Oncology*.

[48] Kwa M., Makris A., Esteva F. J. (2017). Clinical utility of gene-expression signatures in early stage breast cancer. *Nature Reviews Clinical Oncology*.

[49] Gao J., Aksoy B. A., Dogrusoz U. (2013). Integrative analysis of complex cancer genomics and clinical profiles using the cBioPortal. *Science Signaling*.

